# Optimal cut-off for obesity and markers of metabolic syndrome for Ethiopian adults

**DOI:** 10.1186/s12937-018-0416-0

**Published:** 2018-11-22

**Authors:** Makeda Sinaga, Meron Worku, Tilahun Yemane, Elsah Tegene, Tolassa Wakayo, Tsinuel Girma, David Lindstrom, Tefera Belachew

**Affiliations:** 1 Human Nutrition Unit, Faculty of public Health, Jimma University, PO.BOX: 378, Jimma, Southwest Ethiopia; 2 College of Health Sciences, Wolkite University, Welkite, Ethiopia; 3 Faculty of Health Sciences, Department of Laboratory Sciences, Jimma University, Jimma, Ethiopia; 4 Department of Internal Medicine, Faculty of Medicine, Jimma University, Jimma, Ethiopia; 5 Department of Paediatrics and Child Health, Faculty of Medicine, Jimma University, Jimma, Ethiopia; 6 Population Studies Centre, Brown University, Providence, USA

**Keywords:** Obesity, Cut-off, Metabolic Syndrome, Ethiopia

## Abstract

**Background:**

Metabolic syndrome (MetS) is defined as the presence of central obesity plus any two of the following markers: high triglycerides (> 150 mg/dl), low high density lipoprotein (HDL) cholesterol < 40 mg/dl in men and < 50 mg/dl in women, hypertension (blood pressure > 130/85 mmHg or use of antihypertensive medication), high fasting blood glucose (> 100 mg/dl or use of treatment for diabetes mellitus). Since recently, metabolic syndrome and obesity have become emerging problems of both low and middle income countries, although they have been the leading cause of morbidity and mortality in high income countries for the past decades. It has been indicated that the international anthropometric cut-off for detecting obesity is not appropriate for Ethiopians. This study developed optimal cut off values for anthropometric indicators of obesity and markers of metabolic syndrome for Ethiopian adults to enhance preventive interventions.

**Methods:**

A total of 704 employees of Jimma University were randomly selected using their payroll as a sampling frame. Data on socio-demographic, anthropometry, clinical and blood samples were collected from February to April 2015. Receiver Operating Characteristic Curve analyses were used to determine optimal anthropometric cut-off values for obesity and markers of the metabolic syndrome. WHO indicators of obesity based on body fat percent (> 25% for males and > 35% for females) were used as binary classifiers for developing anthropometric cut-offs. Optimal cut-off values were presented using sensitivity, specificity and area under the curve.

**Results:**

The optimal cut-off for obesity using body mass index was 22.2 k/m^2^ for males and 24.5 kg/m^2^ for females. Similarly, the optimal waist circumference cut-off for obesity was 83.7 cm for males and 78.0 cm for females. The cut-off values for detecting obesity using waist to hip ratio and waist to height ratio were: WHR (0.88) and WHtR (0.49) for males, while they were 0.82 and 0.50 for females, respectively. Anthropometric cut-off values for markers of metabolic syndrome were lower compared to the international values. For females, the optimal BMI cut-offs for metabolic syndrome markers ranged from 24.8 kg/m^2^ (triglycerides) to 26.8 kg/m^2^ (fasting blood sugar). For WC the optimal cut-off ranged from of 82.1 cm (triglyceride) to 96.0 cm(HDL); while for WHtR the optimal values varied from 0.47(HDL) to 0.56(fasting blood sugar). Likewise, the optimal cut-offs of WHR for markers of metabolic syndrome ranged from 0.78(fasting blood sugar) to 0.89(HDL and blood pressure). For males, the optimal BMI cut-offs for metabolic syndrome markers ranged from 21.0 kg/m^2^ (HDL) to 23.5 kg/m^2^ (blood pressure). For WC, the optimal cut-off ranged from 85.3 cm (triglyceride) to 96.0 cm(fasting blood sugar); while for WHtR the optimal values varied from 0.47(BP, FBS and HDL) to 0.53(Triglyceride). Similarly, the optimal cut-offs of WHR form markers of metabolic syndrome ranged from 0.86(blood pressure) to 0.95(fasting blood sugar).

**Conclusion:**

The optimal anthropometric cut-offs for obesity and markers of metabolic syndrome in Ethiopian adults are lower than the international values. The findings imply that the international cut-off for WC, WHtR, WHR and BMI underestimate obesity and metabolic syndrome markers among Ethiopian adults, which should be considered in developing intervention strategies. It is recommended to use the new cut-offs for public health interventions to curb the increasing magnitude of obesity and associated metabolic syndrome and diet related non-communicable diseases in Ethiopia.

## Background

Obesity and metabolic syndrome are emerging problems of both low and middle income countries since the past few decades. Metabolic syndrome (MetS) also known as insulin resistance syndrome (syndrome ‘X’) is a constellation of interrelated clinical manifestations characterized by the presence of three or more of the five criteria [1–3]. According to International Diabetes Foundation, the criteria used for the diagnosis of metabolic syndrome includes central obesity (defined as waist circumference with ethnicity specific values) plus any two of the following four: elevated triglycerides(> 150 mg/dl), low high density lipoprotein (HDL) cholesterol < 40 mg/dl in men and < 50 mg/dl in women, hypertension (blood pressure > 130/85 mmHg or use of antihypertensive medication), elevated fasting blood glucose (> 100 mg/d or use of treatment for diabetes mellitus) [1]. Globally, the prevalence of MetS ranged from 10 to 50% [4, 5]. According to WHO, body mass index(BMI) cut-offs > 30 kg/m^2^ is used for defining obesity [5, 6]. Similarly, the definition of central adiposity is waist circumference > 94 cm for men and > 80 for women [7]. Both obesity and metabolic syndrome are highly associated with the development of chronic non-communicable diseases (NCDs). Currently, low income countries are witnessing epidemiological transition from infectious communicable diseases to chronic non-communicable diseases due to changes in the life styles, rapid urbanization and diminishing levels of physical activity [8, 9].

The global prevalence of chronic NCDs is on the rise, with the majority of the increase occurring among populations in developing countries [10]. In low-and middle-income countries, the morbidity burden of NCDs reaches nearly as high as 80%, being the most frequent causes of death in most countries, except in Africa [9].

In Sub-Saharan Africa including Ethiopia, obesity and other markers of metabolic syndrome are emerging problems of public health significance [11]. According to WHO, chronic non-communicable diseases related to obesity will exceed that of infectious diseases in Sub-Saharan Africa by 2030 [9, 12]. This implies that while infections and infestations are still a major health burden in these countries, non-communicable diseases have also become significant problems [11, 13], making the countries grapple with a double burden of diseases.

In Addis Ababa, 46.0% of men and 31.0% of women were pre-hypertensive; while 15.6% of men and 10.8% of women had stage one hypertension in 2011 [14]. This risk of developing metabolic syndrome is rising gradually. People with metabolic syndrome have three times higher risk of suffering a heart attack or stroke and twice the risk of dying from such an event compared with people without the syndrome [3]. To design life style modification interventions for tackling these problems and evaluate their impacts, a cost effective, valid and reliable indicator is critically important. Anthropometric measurements such as body mass index, waist circumference, waist to hip circumference ratio and waist circumference to height ratio are simple, most practical and widely used markers of obesity and metabolic syndromes in such setups, where advanced facilities are non-existent [15–17]. Early detection and prevention of MetS is critical for reducing the burden of non-communicable diseases [18, 19].

However, the above cut-off points for defining both obesity and metabolic syndrome are set based on the Caucasian population and there is an increasing body of evidence that the relation between BMI, body fat percent distribution differs across populations [20]. In particular, for the same level of body fat, age, and gender, BMIs of Ethiopians was 4.6 kg/m^2^ lower compared to Caucasians, showing an underestimation of the level of obesity among Ethiopians [21].This substantiates the prevailing argument on the need for developing population specific BMI and other anthropometric cut-off values. For instance, several epidemiologic studies in Asian populations showed that Asians have higher amounts of body fat at lower waist circumferences than do western populations perhaps leading to the greater prevalence of cardiovascular disease risk factors at lower waist circumference(WC) in Asian populations than in western populations [22, 23]. A similar finding was reported among Ethiopians [21].

Although the magnitude of metabolic syndrome is significantly rising both in developed and developing nations [9, 12], awareness and attention given to early detection of obesity and metabolic syndrome is not adequate in Ethiopian context. Additionally, the prevalence of chronic non-communicable disease is also higher in individuals with lower BMI and other anthropometric parameters indicating the fact that the international anthropometric cut-off is not appropriate for Ethiopians [21]. There is no data on appropriate anthropometric indicators for early detection of obesity and metabolic syndrome in Ethiopia. Therefore, this study is intended to develop optimal cut off values for anthropometric indicators of obesity and markers of metabolic syndrome among Ethiopian adults.

## Methods and materials

The study was conducted among employees of Jimma University located 357 km southwest of Addis Ababa. Jimma University has eight colleges and two institutes training various professionals. There are a total of 5444 workers of which 1341 were academic staff, while the rest were administrative staff. The study was conducted from February to April 2015. Sample size was calculated using sensitivity estimation formula [24] taking prevalence of the most common component of metabolic syndrome (abdominal obesity) of 19.6% among Ethiopian adults (14), margin of error of 5%, a confidence level of 95% and an anticipated sensitivity(SN) of 90%.

$$ \mathrm{n}=\frac{{\left(\mathrm{Z}\ \upalpha /2\right)}^2\mathrm{SN}\ \left(1\hbox{-} \mathrm{SN}\right)}{{\mathrm{d}}^2\left(\mathrm{p}\right)} $$

$$ \mathrm{n}=\frac{(1.96)^20.9(0.1)\ }{(0.05)^2(0.196)}=705 $$

All administrative and academic staff of Jimma University who were actively working at a time of the study were included in the study. Workers who had physical disability including deformity (Kyphosis, Scoliosis, and limb defect), pregnant women were excluded from the study.

A gender stratified simple random sampling was used to select the study participants using proportional to size (PPS) allocation. Sampling frame obtained from JU human resource office based on payroll and computer generated random number were used to select study participants.

### Measurements

Data were collected using WHO STEPS Questionnaire [25] adapted to the local context. A stepwise approach to collect socio-demographic data, anthropometric measurements, clinical measurements, body composition and laboratory analyses of lipid profile and fasting blood glucose level was done. The data were collected by five clinical nurses who were recruited based on their qualification and prior experience of data collection. The data collectors were trained for five days before the actual data collection on interviewing approach, anthropometric measurement and data recording. All the measurements and interviews were done under close supervision of the research team.

### Anthropometry

Height of the study participants was measured to the nearest 0.1 cm using a stadimeter (seca Germany) with the subjects positioned at the Frankfurt Plane and the four points(heel, calf, buttocks and shoulder) touching the vertical stand and their shoes taken off. Before starting the neasurement, the stadiometer was checked using calibration rods. Weight was measured using an electric powered digital scale connected to the plethysmograph (BodPod) to the nearest 0.1 kg with the subjects wearing light closes and shoes taken off. The validity of the scale was checked using an object of a known weight every morning and between the measurements.

Waist circumference was measured at the midway between the lowest costal margin at the midclavicular line and the anterior superior iliac spine using fixed tension tape. Hip circumference was measured at the level of the greater trochanter of the femur with the subjects wearing a pant. All anthropometric measurements were done in triplicate and the average value were used for further analyses. Standardization exercise was done to reduce inter-observer error. Body mass index (BMI) was calculated as the weight in kg divided by height in meters squared (kg/ m^2^).

### Clinical examination

Blood pressure was measured in triplicate using Aneroid Sphygmomanometer with small, medium and large cuff size [26], as fit to the subjects, after 5 min of rest. The subsequent measurements were done 5 min apart. In accordance with the WHO recommendation the mean systolic and diastolic blood pressures were considered for analyses.

### Body composition analyses

Body fat percent was measured using air displacement plethysmography (ADP) (life Measurements) [27, 28] with the subjects wearing only a standardized tight pant after calibration of the equipment before the measurements. Subjects who had longer hair were made to wear swimming cap. Body fat percent was obtained as a print out from the machine and digitally 2 min after each measurement. The study participants were told to come without eating or drinking within 2 h of the measurement. A WHO cut-off for obesity based on body fat percent > 25% for males and > 35% [29–32] for females were used as gold standard binary classifier for obesity to determine anthropometric cut-off values using Receiver Operating Characteristics Curve Analyses.

BMI cut-offs for fitness, athletic and essential body fat percent were developed using the body fat charts of American Council on Exercise (ACE) cited in [33]. The subjects were categorized into the following five groups based on their body fat percent:

- ***For females***: ***Essential Fat*** *(10–13%),* ***Athletes*** *(14–20%),* ***Fitness*** *(21–24%),* ***Average*** *(25–31%) and* ***Obese*** *(32% or higher).*
- ***For males*** *:* ***Essential Fat*** *(2–5%),* ***Athletes*** *(6–13%),* ***Fitness*** *(14–17%),* ***Average*** *(18–24%) and* ***Obese*** *(25% or higher).*

BMI cut-offs for defining the different levels of nutritional status were determined as follows. For severe chronic energy deficiency, the values below the upper range of essential body fat percent according to the American Council on Exercise (ACE) classification cited in [33] for males (5%) and for females (13%) were used to generate BMI cut-off. To determine BMI cut-off for defining the normal body fat percent, we used the body fat percent range suggested by Gallagher et al (2000) [34] for males (8–20%) and for females (21–33%).

For mild to moderate chronic energy deficiency, the lower values for normal body fat percent of males (8%) and females (21%) were used to define the corresponding BMI cut-off. The cut-off values for overweight were determined as a range between the upper value [34] for normal body fat percent (20% for males) and (33% for females) and WHO’s [29–32] body fat percent cut-off for defining obesity (25% for males and 35% for females, respectively).

### Lipid profile and blood glucose level

The laboratory parameters were determined according to the standard operating procedures. Five 5 ml venous blood was collected to determine participants’ fasting blood glucose level and lipid profiles. The subjects were instructed to come for laboratory examination after an overnight fasting. Fasting blood sugar was determined using Humastar within two hours of collection in Jimma University specialized hospital (JUSH) at JUCAN project laboratory. Serum was analyzed to determine lipid profile including total cholesterol, high density lipoprotein(HDL) and triglycerides(TG) using Humastar 80 machine in star III laboratory of Mettu Karl Hospital. Low density lipoprotein level was determined using Freidwald formula [35] as follows: $\mathrm{LDL}-\mathrm{C}\left(\frac{\mathrm{mg}}{\mathrm{dl}}\right)=\mathrm{Total}\ \mathrm{cholesterol}-\left[\mathrm{HDL}-\mathrm{C}+\left(\mathrm{Triglycerides}\div 5\right)\right]$.

All laboratory values were determined by a laboratory technologist who does not know the participants history or other measurements. Lipid profiles and fasting glucose concentrations were reported as mg/dl.

To ensure data quality, pilot-test was done on the study participants who were not included in the main study as they were not selected randomly. Data collectors were trained on the objective of the study, data collection tool and the semantics of each variable on the questionnaire. The data collectors were four masters’ nutrition students, three master holder laboratory technologists and five experienced degree holder nurses. There was also demonstration and practical session on interviewing and anthropometric measurements. Standardization exercise was done on anthropometric measurements to reduce inter observer error. ADP was calibrated every morning using an object of known weight and between the measurements. Furthermore, the weight scale indicator was checked against zero reading after weighing every individual. The measurements were also randomly rechecked during data collection.

### Data processing and analysis

First the data were checked for completeness and consistency and then double entered into EPI data software version 3.1 to check clerical errors. Then, the data were exported to SPSS for windows version 20 program for analyses. The data were cleaned by checking outliers and missing values. Descriptive analysis of the background characteristic was performed. Normality was checked for continuous variables.

Receiver operating characteristic curve (ROC) was developed using obesity determined based on ADP determined body fat percent(> 25% for males and > 35% for females) as a binary classifier for identifying the optimal cut-off values of all continuous anthropometric, laboratory and clinical variables. Area under curve (AUC), sensitivity, specificity and Youden’s index values were determined for normal ranges, obesity and indicators of MetS markers. ROC curves were used to determine the discriminatory power of anthropometric indices in distinguishing adults with high blood pressure, high fasting blood glucose, dyslipidaemia and markers of metabolic syndrome. The optimal cut-off values were defined as a point on the curve where Youden’s index (defined as: sensitivity + specificity – 1), is maximum [36].

## Results

A total of 704 participants were enrolled into the study of which 397 were females and 307 were males. The mean (sd) age of the study participants was 36.5(9.2) years for females and 34.7(9.5) years for males. The maximum and minimum ages were 20 and 60 years for males and 19 and 60 years females, respectively. The largest proportion (36.2%) was Oromo by Ethnicity followed by Amhara (30.3%) and Dawero(8%).

The mean (sd) body Mass index was 25.3(5.1) kg/m^2^ for females and 22.5(3.9) kg/m^2^ for males. The mean (sd) height and weight were 157.2(8.5) cm and 62.3(12.9), respectively for females; while it was 171.8(13.4) cm and 67.0(11.7) kg for males, respectively. The mean (sd) waist circumference was 83.6(14.7) cm for females and 84.1(11.4) cm for males; while the mean (± sd) body fat percent (BF %) was 38.47(10.05) for females and 23.86(9.16) for males (Table 1).

**Table 1:** Background characteristics of Ethiopian adults who participated in the study

Variables	Sex		n
	Female	Male	
	No. (%)	No. (%)	
Ethnicity			
Oromo	106(41.6)	149(58.4)	255
Amhara	140(65.7)	73(34.3)	213
Gurage	22(57.9)	16(42.1)	38
Kefa	38(76.0)	12(24.0)	50
Others (Sidama,Wolaita,Tigre)	20(41.7)	28(58.3)	48
Dawro	40(70.2)	17(29.8)	57
Yem	31(72.1)	12(27.9)	43
	Mean(sd), *n* = 397	Mean(sd), *n* = 307	
Age	36.49(9.20)	34.73(9.49)	
BMI	25.29(5.13)	22.49(3.91)	
Height	157.15(8.53)	171.77(13.35)	
Weight	62.34(12.93)	66.99(11.73)	
Waist Circumference	83.63(14.70)	84.05(11.42)	
Hip Circumference	98.63(11.67)	93.04(16.37)	
Body fat percent (BF%), mean(±sd)	38.47(10.05)	23.86(9.16)	

*BMI* Body mass index, *sd* standard deviation

### Optimal cut-off for different anthropometric measurements to diagnose obesity

From the ROC analyses, the optimal cut-offs for diagnosing obesity using different anthropometric parameters are presented in Table 2. The optimal cut-offs for defining obesity using BMI were 22.2 k/m^2^ for males and 24.5 kg/m^2^ for females. Similarly, the optimal cut-offs of for defining obesity using waist circumference were 83.7 cm for males and 78.0 cm for females; while the cut-offs for diagnosing obesity using waist to hip circumference ratio were 0.88 for males and 0.82 for females. Likewise, the optimal cut-offs for waist to height ratio to diagnose obesity were 0.49 for males and 0.50 for females.

**Table 2:** Anthropometric cut-offs for diagnosing obesity among Ethiopian adults

Anthropometric indicators	AUC(95%CI)	Optimal cut-off	Sensitivity (%)	Specificity (%)	Yuden index (%)	Std.err	*P*
Males							
BMI	0.922(0.891–0.954)*	22.2	88.0	87.7	76.0	0.016	< 0.001
WC	0.945(0.921–0.969)*	83.7	84.9	91.6	76.5	0.012	< 0.001
WHR	0.854(0.812–0.897)*	0.88	85.5	72.9	58.4	0.022	< 0.001
WHtR	0.952(0.931–0.973)*	0.49	86.0	90.0	76.0	0.011	< 0.001
Females							
BMI	0.949(0.930–0.968)*	24.5	80.0	95.6	75.6	0.010	< 0.001
WC	0.904(0.872–0.936)*	78.0	84.0	87.0	70.0	0.016	< 0.001
WHR	0.723(0.668–0.777)*	0.82	73.0	74.0	47.0	0.028	< 0.001
WHtR	0.913(0.873–0.943)*	0.50	86.0	85.0	71.0	0.015	< 0.001

*BMI* body mass index, *WC* waist circumference, *WHR* weight to hip ratio, *WHtR* weight to height ratio, *TG* Triglyceride, *HDL* High density lipoprotein, *FBS* Fasting Blood press sure, *AUC* area under the curve

*P < 0.001

**Fig. 1:**
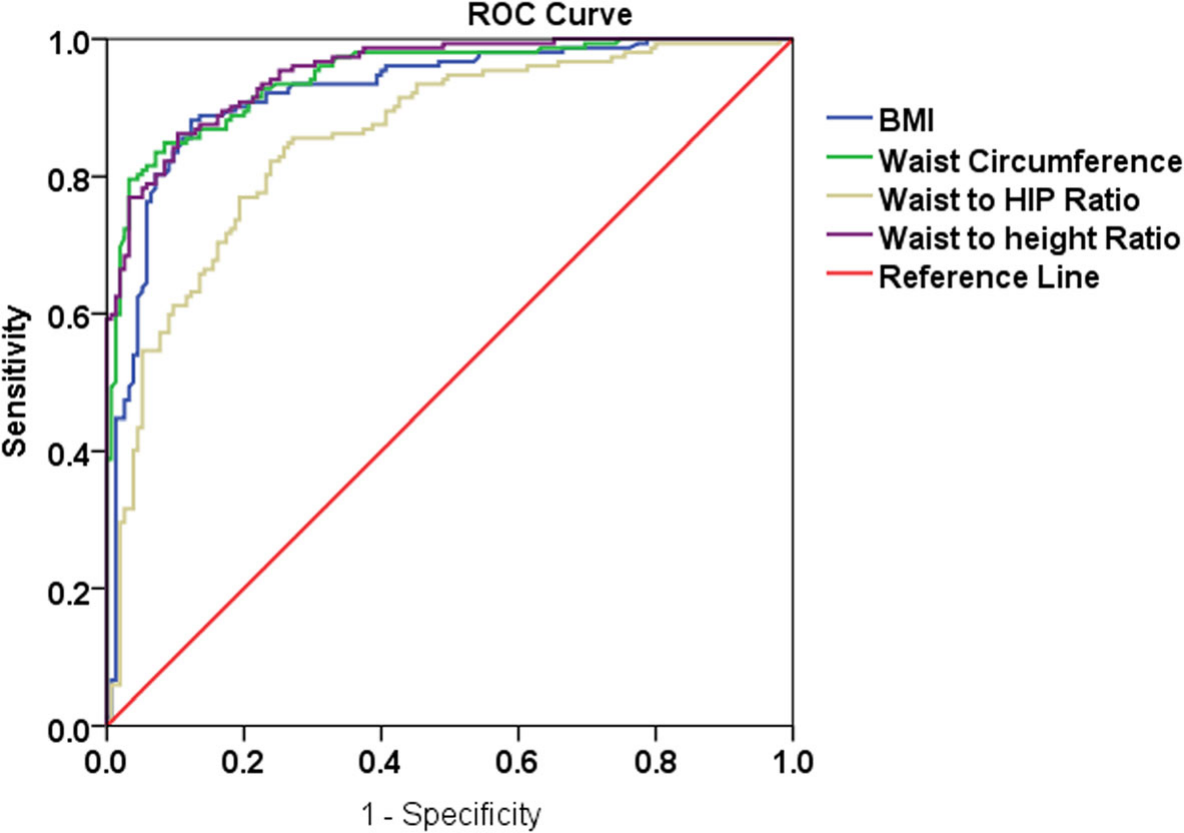
Receiver operating characteristics curve showing the performance of different anthropometric measurements in detecting obesity among Ethiopian adult males. WC=Waist Circumference, BMI=Body Mass index, WHR = Waist to Hip Ratio, WHtR = Waist to Height Ratio

Anthropometric parameters that showed higher sensitivity and specificity in predicting obesity among males were BMI, waist circumference, and waist to height ratios. A presented in Fig. 1, the ROC curve showed that BMI (AUC = 0.922; 95%CI: 0.891–0.954), waist to height ratio (AUC = 0.952, 95%CI: 0.931–0.973) and waist circumference (AUC = 945, 95%CI: 0.921–0.969) had better sensitivity and specificity with largest areas covered under the curve, while waist to hip ratio had a relatively lower sensitivity and specificity (AUC = 0.854, 95%CI: 0.812–0.897).

**Fig. 2:**
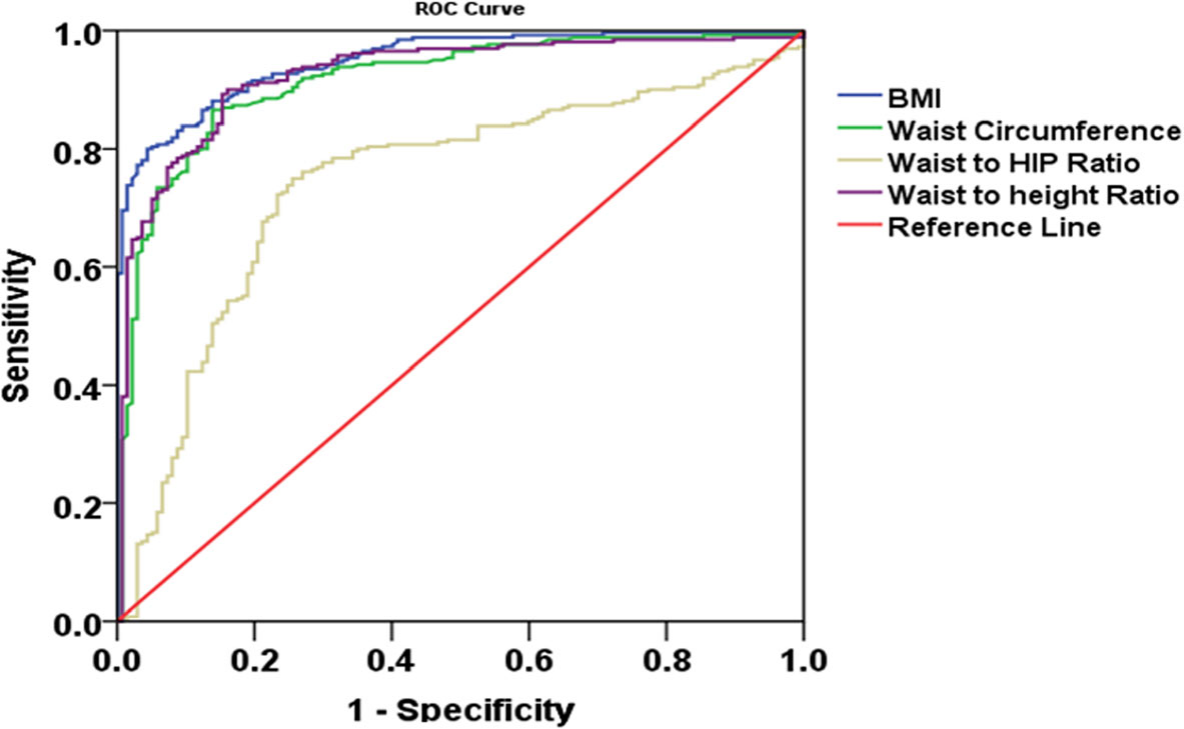
Receiver Operating Characteristics Curve showing the performance of different anthropometric parameters in detecting obesity among Ethiopian adult Females. WC=Circumference, BMI=Body Mass index, WHR = Waist to Hip Ratio, WHtR = Waist to Height ratio

Similarly, the ROC curve for females showed that BMI (AUC = 0.949, 95%CI: 0.930–0.968), waist to height ratio (AUC = 0.913, 95%CI: 0.873–0.943) and waist circumference (AUC = 0.904, 95%CI: 0.872–0.936) had better sensitivity and specificity with largest areas under the curve covered, while waist to hip ratio had relatively lower areas covered under the ROC curve(AUC = 0.723, 95%CI: 0.668–0.777), Fig. 2.

### Optimal BMI cut-off for fitness, athletic and essential body fat percent

Tables 3 and 4 present the optimal BMI cut-off indicating fitness, athletic and essential body fat percentages among Ethiopian adults. Accordingly, for males the optimal BMI cut-off to determine body fat percent of fitness was 21.5 kg/m^2^ (AUC = 0.88, 95% CI: 0.84, 0.92); while it was 20.9 kg/m^2^ (AUC = 0.86, 95%CI: 0.81, 0.91) and 15.5 kg/m^2^ (AUC = 0.99, 95%CI: 0.98, 1.00) for athletic and essential body fat percents, respectively.

**Table 3:** BMI cut-offs for fitness, athlete and essential body fat percent for Ethiopian adults

Sex	Level of BF%	AUC(95%CI)	Optimal BMI cut-off	Sensitivity	Specificity	Youden index	Satd.err	*P*
Male	Fitness	0.88(0.84, 0.92)	21.5	92.0	73.4	65.5	0.021	*< 0.001*
	Athletic	0.86(0.81, 0.91)	20.9	90.0	69.6	59.6	0.026	*< 0.001*
	Essential fat	0.99(0.98, 1.00)	15.5	100.0	99.0	99.0	0.05	*0.09*
Female	Fitness	0.94(0.91, 0.97)	22.0	100.0	82.0	77.0	0.015	*< 0.001*
	Athletic	0.93(0.90, 0.97)	21.9	100.0	75.9	75.9	0.017	*< 0.001*
	Essential fat	0.99(0.84, 1.00)	20.6	100.0	82.5	82.5	0.012	*0.042*

The subjects were categorized into the following three groups based on their body fat percent according to American Council on Exercise (ACE) [33]: For females: Essential Fat (10–13%), Athletes (14–20%), Fitness (21–24%). For males: Essential Fat (2–5%), Athletes (6–13%), and Fitness (14–17%)

*AUC* Area under the curve, *BF%* body fat percent

**Table 4:** BMI cut-offs for diagnosing overweight and undernutrition (Chronic energy deficiency) among Ethiopian adults

Nutritional status	Optimal BMI cut-off (kg/m^2^)	
	Males	Females
Obese^a^	> 22.2	> 24.5
Overweight	21.6–22.2	23.1–24.5
Normal^b^	18.3- 21.5	21.9–23.0
Mild to Moderate Chronic Energy deficiency	15.5–18.2	20.6–21.8
Severe Chronic Energy Deficiency	< 15.5	< 20.6

^a^Obesity cut-off was generated from the ROC analyses using WHO cut-off for obesity based on body fat percent > 25% for males and > 35% [29–32] as binary classifier

^b^The normal BMI cut-off was determined as a range between Chronic energy deficiency and Overweight

Similarly, for females, the optimal BMI cut-off indicating body fat percent of fitness was 22.0 kg/m^2^ (AUC = 0.94, 95%CI: 0.91, 0.97); while it was 21.9 kg/m^2^(AUC = 0.93, 95%CI: 0.90, 0.97) for athletic and 20.6 kg/m2 (AUC = 0.99,95%CI:0.84,1.00) for essential body fat .

### Optimal BMI cut-off for overweight and chronic energy deficiency

Based on the above analyses the cut-off values for the different ranges of nutritional status were determined for Ethiopian adults. The cut-off values for overweight were 21.6–22.2 kg/m^2^ for males; while it was 23.1–24.5 kg/m^2^ for females. The normal range for BMI was 18.3–21.5 kg/m^2^ for males, while it was 21.9–23.0 kg/m^2^ for females.

The cut-off values for the other extreme form of malnutrition (Chronic energy deficiency) among Ethiopian adults were determined as follows. For males BMI 15.5–18.2 kg/m^2^ was an indicator of mild to moderate chronic energy deficiency; while BMI < 15.5 kg/m^2^ was an indicator of severe chronic energy deficiency. Similarly, the cut-off values for chronic energy deficiency in females were as follows: BMI 20.6–21.8 kg/m^2^ was an indicator of mild to moderate chronic energy deficiency while BMI < 20.6 kg/m^2^ was an indicator of severe chronic energy deficiency(CED).

### Optimal anthropometric cut-offs for the markers of metabolic syndrome

The cut-off values for different anthropometric laboratory and clinical parameters for detecting the different markers of metabolic syndrome were also determined using ROC analyses (Table 5).

**Table 5:** Optimal cut off point for components of metabolic syndrome among JU employees from February–April 2015

Markers of MetS	Anthropometric index	AUC (95%CI)	Optimal cut-off	Sensitivity%	Specificity%	Yuden index	Std.err
Females							
BP(≥130/85 mmHg)	BMI	0.61(.54,.68)	26.2	59.0	60.0	19.0	0.036
	WHtR	0.64(.57,.71)	0.51	77.0	47.0	24.0	0.035
	WC	0.62(.55,.69)	93.0	43.0	79.0	22.0	0.036
	WHR	0.59(.52,.66)	0.89	43.0	76.0	19.0	0.037
FBS ≥ 100 mg/dl	BMI	0.59(.53,.65)	26.8	48.0	70.0	18.0	0.031
	WHtR	0.57(.51,.63)	0.56	45.0	69.0	13.0	0.031
	WC	0.58(.52,.64)	83.1	56.0	58.0	14.0	0.031
	WHR	0.52(.46,.58)	0.78	81.0	28.0	9.0	0.030
TG ≥150 mg/dl	BMI	0.60(.53,.66)	24.8	64.0	54.0	18.0	0.032
	WHtR	0.59(.53,.65)	0.53	59.0	59.0	18.0	0.032
	WC	0.59(.53,.66)	82.1	65.0	53.0	18.0	0.033
	WHR	0.57(.50,.63)	0.88	43.0	73.0	15.0	0.033
HDL < 50 mg/dl	BMI	0.52(.41,.63)	25.0	63.0	52.0	15.0	0.055
	WHtR	0.53(.42,.65)	0.47	37.0	78.0	15.0	0.057
	WC	0.49(.38,.60)	96.0	89.0	19.0	7.4	0.056
	WHR	0.51(.41,.61)	0.89	85.0	26.0	11.0	0.051
Males							
BP(≥130/85 mmHg)	BMI	0.69(.61, .77)	23.5	68.0	65.0	32.0	0.039
	WHtR	0.71(.64,.79)	0.47	87.0	50.0	36.0	0.038
	WC	0.74(.62, .85)	89.22	90.9	58.0	49.0	0.059
	WHR	0.70(.63, .78)	0.86	90.0	47.0	38.0	0.039
FBS ≥ 100 mg/dl	BMI	0.68(.62, .74)	21.1	80.0	55.0	34.0	0.031
	WHtR	0.69(.63, .75)	0.47	78.0	52.0	30.0	0.031
	WC	0.67(.57, .77)	96.0	38.0	96.0	34.0	0.050
	WHR	0.66(.59, .72)	0.95	45.0	82.0	27.0	0.033
TG ≥150 mg/dl	BMI	0.68(.62, .75)	22.5	71.0	63.0	34.0	0.032
	WHtR	0.71(.65, .77)	0.53	54.0	81.0	34.0	0.031
	WC	0.74(.69, .80)	85.3	69.0	72.0	41.0	0.030
	WHR	0.71(.65, .77)	0.90	73.0	62.0	35.0	0.030
HDL < 40 mg/dl	BMI	0.53(.43, .63)	21. 0	49.0	64.0	12.0	0.051
	WHtR	0.57(.47, .66)	0.47	56.0	62.0	17.0	0.049
	WC	0.54(.43, .65)	89.3	60.0	53.0	13.0	0.054
	WHR	0.60(.51, .69)	0.90	69.0	55.0	24.0	0.045

*BMI* body mass index, *WC* waist circumference, *WHR* weight to hip ratio, *WHtR* weight to height ratio, *TG* Triglyceride, *HDL* high density lipoprotein, *FBS* Fasting Blood Sugar, *AUC* Area Under the Curve, *MetS* Metabolic Syndrome

Accordingly, for females, the cut-off values for high blood pressure (> 130/85mmhg) were BMI (26.2kgm^2^), WHtR (0.51), WC (93 cm) and WHR (0.89). The cut-off values for high fasting blood sugar were (FBS) > 100 mg/dl) was 26.8 kg/m^2^ for BMI; 0.56 for WHtR, 83 cm for WC and 0.78 cm for WHR. The cut-off values for high Triglyceride level (TG ≥ 150 mg/dl) were: BMI (24.8 kg/m^2^), WHtR (0.53), WC (82.1 cm) and WHR(0.88). The cut-off values for detecting low HDL level (< 50 mg/dl) were: BMI (25 kg/m^2^), WHtR (0.47), WC(96 cm) and WHR(0.89).

Likewise, anthropometric cut-off values for indicating the presence of components of metabolic syndrome in males are presented in Table 5. The cut-off values for high blood pressure (> 130/85mmhg) were BMI (23.5kgm2), WHtR (0.47), WC (89.22 cm) and WHR (0.86). The cut-off values for High fasting blood sugar (FBS > 100 mg/dl) were: BMI (21.1 kg/m^2^), WHtR (0.47), WC (96 cm) and WHR (0.95). The cut-off values for high Triglyceride level(TG ≥ 150 mg/dl) were: BMI(22.5 kg/m^2^), WHtR(0.53), WC(85.3 cm) and WHR(0.90) while the values for detecting low HDL level (< 40 mg/dl) were: BMI (21.0 kg/m^2^), WHtR(0.47), WC(89.3 cm) and WHR(0.90). 

## Discussion

We found that the optimal BMI cut-off for defining obesity Ethiopian adults were 22.2 kg/m^2^ for males and 24.5 kg/m^2^ for females, which is close to the report of a study done among Indonesian adults, that indicated a BMI cuts-offs of 21.9 kg/m^2^ for males and 23.6 kg/m^2^ for females [37]. Similarly, a study in China reported that the cut-off values for defining overweight using BMI were 22.5 kg/m^2^ for males and 23.5 kg/m^2^ for females [38], which is closer to our findings. Similar lower cut-off values were reported for defining obesity and components of metabolic syndrome in other Asian countries including Taiwan [39], Korea [40] Malaysia, and China [38] and risk of coronary heart disease Malaysia [41].The optimal cut-off values for BMI, waist circumference, waist to height ratio and waist to hip ratios were lower than the cut-off values set based on the Caucasian population indicating the fact the European cut-off suggested by WHO [7] for countries that do not have their population specific data is not appropriate for Ethiopian adults. Our finding confirmed that existing body of literature on the fact that using the European cut-off for predicting adiposity in Ethiopian population would lead to underestimation and misclassifies the risk of metabolic syndrome significantly [21]. According to WHO recommendations, the BMI threshold for increasing disease risk in Caucasian population is 25 kg/m^2^ for both men and women [7]. This value was suggested to be 23 kg/m^2^ in Asian men and women [42]. Cut-points developed by our study are lower than those recommended for Caucasians and Asians [6, 7].

The reason might be due to the fact that Ethiopians have slender body build which has high body fat with lower BMI, such that with the same level of BMI, age, and gender, body fat percent of Ethiopians was 10% higher compared to Caucasians [21]. A similar disparity between BMI and body fat percent has been reported between Indians and Caucasians [41].

Similarly, the cut-off values for detecting obesity using waist circumference, waist to hip ratio and waist to height ratio were WC (83.7 cm), WHR (0.88) and WHtR (0.49) for males, while they were WC (78.0 cm), WHR(0.82) and WHtR (0.50) for females. The cut-offs developed from this study were a little higher than the corresponding findings from Indonesian adults [37], which were: WC (76.8 cm), WHR (0.86), and WHtR (0.48) for males, while they were 71.7 cm, 0.77 and 0.47 for females indicating that their cut-off values are lower than our findings. This disparity might be due to methodological differences in the determination of body fat percent.

Despite this difference, the international cut-off resulted in a very high underestimation of the predicted body fat percent as compared to the measured body fat percent among Ethiopians and Indonesians [21]. The fact that universal cut-off BMI points for obesity are not appropriate has also been indicated [43]. Measured body fat percent was reported to be underestimated by BMI based Caucasian prediction equation among Ethiopians, Malaysian and Thai [21].

Similar to our findings, waist circumference and waist to height ratio were strong predictors of obesity as reported by other studies [37, 38, 44]. In this study, anthropometric measures such as WC and WHtR were good predictors of body fat percent in males; while BMI was observed to be better predictors in females, which is consistent with the findings of another study [45].The fact that anthropometric measures of body fat including WC, BMI, WHtR are strongly related to one another than overall body fat percent and the need for having specific cut-off values for sex and age has also been suggested [46].

In this study, waist to hip circumference ratio was poor predictors of obesity in both sexes especially in female adults compared to other parameters. A study with a similar finding suggested that it should not be used as a surrogate marker of abdominal visceral fat especially in women [47].

Our findings showed that anthropometric cut-off values for predicting the markers of metabolic syndrome were lower compared to the international cut-off.

For females, the optimal BMI cut-offs for metabolic syndrome components ranged from 24.8 kg/m^2^ (Triglycerides) to 26.8 kg/m^2^ (fasting blood sugar); while for males the optimal BMI cut-offs for metabolic syndrome components ranged from 21.0 kg/m^2^ (HDL) to 23.5 kg/m^2^ (blood pressure). In this study, the normal range for BMI was set to be 18.3 to 21.5 kg/m^2^ for males and 21.9 kg/m^2^ to 23.0 kg/m^2^ for females. The small overlap between the optimal values of two markers of metabolic syndrome 21.0 kg/m^2^ (HDL) and 21.1 kg/m^2^ (FBS) and the upper values of the normal range for BMI (21.5 kg/m^2^) observed in males could be explained by the fact that we used different references for developing optimal cut-offs for fitness, athletic and essential body fat [33] and for defining normal range [34] and obesity [29–32]. However, this overlap is acceptable as some variation in the markers of metabolic syndrome could occur surrounding the upper cut-off values even in the normal range.

The BMI, cut-off values for defining components of metabolic syndrome varied from 26.2 to 27.2 kg/m^2^ in men and from 27.2 to 30.0 kg/m^2^ in women in Jordan [45] and from 25 kg/m^2^ in men to 28 kg/m2 in women in Saudi Arabia [47], which are higher than our findings. This difference might be due to the disparities in the methods used for measuring body fat percent as well the differences in body frame between these populations owing to the racial differences. The Caucasian cut-off has also been shown to underestimated obesity among Ethiopians [21].

Our results also showed that the cut-off values of WC for detecting the components of metabolic syndrome ranged from 82.1 to 96 cm for females and 85.3 to 96 cm for males. Similarly, the cut-off values of WHR for detecting the components of metabolic syndrome for women ranged from 0.78 to 0.89; while that of men ranged from 0.86 to 0.95, which is very close to the reports of other studies [45, 48]. The optimal cut-off values for WC and WHR were 92 cm, 0.89, for men and 87 cm, 0.81 and for women for identifying the risk of metabolic syndrome in Saudi Arabia [48]. Values of WC fall into a wider range (from 88.5 to 91.8 cm in men and from 84.5 to 88.5 cm) in women in Jordan [45]. Similarly, the optimal cut-off points of the different anthropometric measurements including WC, WHR and WHtR for indicating the components of metabolic syndrome were lower for the study population as compared to the international cut-off values suggested by WHO [7].

The new cutoff points developed by this study for BMI, WC and WHtR showed excellent performance in detecting obesity with the areas under the curve being above 0.9, showing that they can be used a simple, user friendly and cost-effective tools for screening obesity [49, 50]. This is given the fact that other methods of body composition measurements are expensive and inaccessible and the international cut-off is inappropriate for Ethiopians. Furthermore, the findings have far reaching practical implication as Ethiopia is aspiring to be a lower middle income country by 2025 [51] and there is a rapid urbanization and change of life styles [52]. The country is already facing double burden of undernutrition and obesity, which is expected to take a sharp turn to the worst scenario given the history of high level childhood stunting experienced in the country [53]. Undernutrition in early life could lead to organ stunting [54] and increased risk of metabolic syndrome. This was indicated to be a fertile ground for the looming prevalence of obesity and chronic non-communicable diseases [54–56].

Recently, an increase in mortality due to chronic non-communicable diseases related to obesity is also reported in Ethiopia [57, 58]. These findings will be essential inputs for the preparatory programs to tackle such upcoming problems in the country.

This study used air displacement plethysmography as a gold standard for determining body fat percent, which makes the cut-offs points valid and reliable [27, 28]. This study purposely used university staff for developing the different cut-offs as it gives a more ethnically representative sample. This might raise the concern of the representativeness of the data to the community. However, this will not be a problem as the samples were drawn randomly with the representation of subjects with different levels of body composition. Moreover, the comparison anthropometric measurements and body fat percent generated by ADP was made within the subjects themselves, making this possibility very thin. Although the study tried to involve multiple ethnicities, some of the smallest ethnic groups were not represented sufficiently. However, we do not expect much difference between the different ethnic groups than what the study sample could represent as most of the differences are related to language and culture; except people in Gambella due to possible differences in sitting height to height ratio. Future research should look into the appropriateness of the cut-off developed in this context. Anthropometric measurements errors are also likely to happen and would make a difference in such analyses. However, this study used the highest precaution in training the data collectors, in the calibration of equipment and standardization of procedure to minimize the possibility of errors. Given this context, we believe that the cut-off values generated by this study are useful tools for promoting public health interventions to prevent obesity and related NCDs in Ethiopia.

## Conclusion

The optimal anthropometric cut-offs for detecting obesity and markers of metabolic syndrome in Ethiopian adults are lower than the international cut-offs. The findings imply that the international cut-off for WC, WHtR, WHR and BMI underestimate obesity and metabolic syndrome markers among Ethiopian adults, which should be considered in developing intervention strategies. To curb the increasing magnitude of obesity and associated metabolic syndrome and NCDs, it is recommended to:

- Design and develop polices targeting early detection of metabolic syndrome, to prepare national advocacy and health information on nutrition programs at population level.
- Promote self-screening at household level by using WC, WHtR and BMI measurement to improve life style of the community as early as possible.
- Shift the focus from treatment approach to preventive approach for chronic diseases by using affordable and sensitive indicators like waist circumference, BMI and WHtR.
- Strengthen early preventive life style modification program based on the revised cutoffs
- Consider the new cutoffs points while preparing guidelines and intervention strategies.

## Abbreviations

ADP: Air Displacement Plethysmography
BMI: Body Mass Index
ROC: Receiver Operating Characteristics Curve
WC: Waist Circumference
WHR: Waist to Hip Ratio
WHtR: Waist to Height Ratio
